# The importance of assigning responsibility during evaluation in order to increase student satisfaction from physical education classes: A structural equation model

**DOI:** 10.1371/journal.pone.0209398

**Published:** 2019-09-10

**Authors:** Marta Leyton Román, Susana Lobato Muñoz, Ruth Jiménez Castuera

**Affiliations:** 1 Sport Studies Center, Rey Juan Carlos University, Madrid, Spain; 2 Faculty of Sport Sciences, University of Extremadura, Cáceres, Extremadura, Spain; 3 Didactic and Behavioural Analysis in Sport Research Group, Faculty of Sport Sciences, University of Extremadura, Cáceres, Extremadura, Spain; Hong Kong Polytechnic University, HONG KONG

## Abstract

Considering the benefits that students report when evaluating physical education classes, the purpose of the present study was to analyse the relationships between the assignment of student responsibility in the evaluation, motivational variables and the satisfaction with the physical education classes, using The Theory of Self-determination as a support method. The sample for this study was 922 students, of both genres and in Compulsory Secondary Education, aged between 14 and 18 years. To carry out the study, the Student's Scale of Responsibility was used in the physical education assessment, the Basic Psychological Needs Measuring Scale, the Percentage Scale for Physical Education Causality and the Satisfaction Scale in Physical Education. The results of the structural equations model revealed a good adjustment to the data. This finding highlights the importance of giving responsibilities to the students in the evaluation process, in order to satisfy the psychological needs of the students and, therefore, self-determined motivation. Additionally, the satisfaction of psychological needs and self-determined motivation increase satisfaction towards physical education classes.

## Introduction

Physical education (PE) has become a framework for many youngsters to carry out physical activities, which then increases their motivation and adherence to do exercise after school [1, 2, 3].

The teacher is one of the main promoters of the practising of physical activities [4], for which their figure is crucial for the students to increase, or not, their level of regular physical activity [5]. However, in PE there are still authors [6, 7] who indicate that, traditionally, teaching has consisted in a pedagogical model of direct instruction.

Research has determined that the teacher’s use of strategies, with positive psychological aspects, such as the increase of students’ intrinsic motivation in PE classes, will allow for the development and consolidation of behaviours related to physical activity [8, 9, 10].

An interrelated set of motivational phenomena, which combine biological, emotional, cognitive and social aspects influence the persistence, intensity and frequency of behaviour. By interacting with each other, they also increase, maintain or decrease this behaviour [11].

The present study focuses on the Theory of Self-Determination (SDT) [12,13]. In the SDT, the motivation is structured through a continuum that encompasses the different degrees of self-determination of behavior, within which they are distinguished [14, 15], from less to more self-determined: the demotivation (lack of interest in the activity), controlled motivation (behaviors that are controlled by external reinforcers), and autonomous motivation (involves performing an activity for the pleasure of practicing it) [16].

In the SDT, the Basic Psychological Needs (BPN) of autonomy (desire to engage in activities by own choice), competence (desire to interact efficiently with the environment to feel competent) and relatedness (desire to feel part of a group) [16, 12]. They constitute the psychological mediators that will influence the three main types of motivation [12,17]. Numerous studies have used BPN as mediators that positively predict more self-determined forms of motivation [18, 19, 20, 21].

From there arises the Hierarchical Model of Motivation (HMM) [22]. According to the HMM, there are three hierarchical levels where the motivation (global, contextual and situational), which can be affected, as some levels are related to others. The model establishes that the social aspects of the environment (background variables) influence motivation, depending on the achievement or not of a series of BPN (autonomy, competence and relatedness), where satisfaction increases the degree of intrinsic motivation (motivational variables) [12, 16] and will lead to positive consequences on a cognitive, affective and behavioural level (consequent variables).

A climate in which responsibility is given to the student will generate positive thoughts about physical activity [23]. Different studies have revealed that when the teacher provides students with autonomy and responsibility, they value more highly the PE classes and their enjoyment also increases [24, 25, 26]. In a recent study [27], with 532 students, guided by the TAD hypothesis, it was concluded that the student profiles of PE classes were mainly autonomous ones.

Evaluation can be considered as an instrument for monitoring and evaluating the results obtained by a student. The teacher can employ a more controlling teaching style, where more importance is given to results than to the learning process, or a teaching style that favours the autonomy of the student, where the student is a participant in their own learning process, using techniques such as self-evaluation, co-evaluation or hetero-evaluation [28]. Given the importance of the process in the achievement of results by the students, in this study, as a prior variable to the motivational variables, the perception of the assigning of student responsibility was used in the evaluation.

According to Hortigüela-Alcalá et al. [28], students take pleasure from being offered different strategies and alternatives to achieve their goals; This, in turn, increases intrinsic motivation towards classes and thereby the likelihood of students exercising outside of the classroom [29, 30]. Studies like that of Yonemura et al. [31], indicated that student participation in evaluation produced an increase in their commitment to learning. In the same way, it revealed the importance of proposing different strategies for evaluation in which student participation is included [32, 33]. Likewise, other authors [34, 35, 36, 37] highlight the importance of PE and sport being directed towards student autonomy and the designation of student responsibilities, claiming that a teaching style that gives subjects the chance to choose, participate and make decisions in classes, will give rise to a more enjoyable participation and an increase in intrinsic motivation [18, 38, 39]. Therefore, students need to be given the opportunity to participate, by being given responsibilities. [40, 41, 42].

Following the HMM, the consequent variable of the present study was satisfaction with PE classes. According to Herrera-Mor et al. [43], the enjoyment that is experienced from an activity, understood as satisfaction in relation to pleasure and well-being, allows for participation to remain throughout time, for a greater adherence and for participation to become an integral part of lifestyle.

Similarly, enjoyment can be understood as the valued sense of the activities carried out in PE classes by the students [44]; and this variable (satisfaction with PE classes) is even related to the obtaining of a better academic qualification [45, 46]. As some studies have indicated, to avoid the abandonment of physical activity, teachers must try to make activities fun and avoid those which are not entertaining [47], thus presenting the teacher with an essential role to play in the development of these activities [48].

In this regard, Ntoumanis [49] explained that when subjects have fun they tend to be intrinsically motivated and give more importance to the subject. Satisfaction with PE classes will be positively related to the satisfaction of BPN as well as to a more self-determined motivation [44, 48].

In the same way, González-Cutre et al. [50] emphasize the importance of the use of motivational strategies through BPN in PE classes, confirming the achievement of positive consequences such as learning, enjoyment and adherence to sports.

Further, Moreno et al. [51], in a sample of 819 students aged between 14 and 17 years, discovered that the most self-determined form of motivation positively predicted the importance given to PE classes and with this, satisfaction with the same.

In relation to satisfaction, motivation and boredom with PE classes, as several studies have shown [10, 52, 53, 54, 55, 56], high levels of self-determination are associated with greater effort, enjoyment, the importance of PE and the development of positive behaviour. In contrast, if motivation is less self-determined, the consequences will be negative, such as boredom in classes [9, 57].

Thus, the objective for this study was to analyse the relationships between the designation of student responsibility in evaluation, motivational variations and student satisfaction with PE classes, through obtaining a model of structural equations.

The theoretical model will facilitate and approach on the strategies of the PE teacher, specifically, the following hypothesis were raised: (1) the assignment of responsibility towards the student will predict, in a positive and significant way, the satisfaction of the BPN of autonomy, competence and relatedness; (2) the satisfaction of the BPN of autonomy, competence and relatedness will predict, in a positive and significant way, the autonomous motivation; (3) the autonomous motivation will predict, in a positive and significant way, student satisfaction with PE classes.

## Material and methods

The study received the approval of the Commission of Bioethics and Biosecurity of the University of Extremadura (Spain) following the guidelines of the Helsinki Declaration. All participants were treated in agreement with the ethical guidelines of the American Psychological Association with respect to participant assent, parent/guardian consent, confidentiality and anonymity. Moreover, informed written consent was obtained from the participants and their parents/guardians.

### Research design

The study carried out was non-experimental, in which the variables described above have not been altered or manipulated, only what occurs with them under natural conditions having been observed [58].

Likewise, it is located within quantitative empirical studies and, within these, it refers to the descriptive study of populations through surveys [59].

### Sample

The study sample was 922 students of both sexes (430 male and 492 female) from compulsory secondary education, more specifically from 3rd and 4th year of secondary school. 12 students were excluded from the study. The exclusion criteria were: not answering most of the questions and unusual response patterns.

The type of sampling that was carried out was intentional. There were 9 schools with a total of 50 classes. Each class was constituted by 18–19 students. The ages of the sample were between 14 and 18 years (M = 14.95, SD = .98).

In Table 1, the distribution of the sample in terms of gender and year-group can be seen.

**Table 1:** Distribution of the sample according to gender and year group.

Year-Group	Male	Female	Total
**3°**	247	265	512
**4°**	183	227	410
**Total**	430	492	922

In Table 2, the distribution of the sample in terms of organisations that participated in the study and year-group is represented.

**Table 2:** Distribution of the sample according to the school and the year-group.

School	3°		4°		Total
	Male	Female	Male	Female	
**School 1**	22	20	16	10	68
**School 2**	19	27	11	3	60
**School 3**	22	36	24	32	114
**School 4**	54	56	47	48	205
**School 5**	29	19	22	32	102
**School 6**	42	43	18	60	163
**School 7**	18	21	0	0	39
**School 8**	41	43	38	35	157
**School 9**	0	0	7	7	14
**Total**	247	265	183	227	922
**Total Year-Group**	512		410		922

### Variables and measurement tools

In this section, the variables present in this investigation are revealed, divided according to the HMM: antecedent, motivational and consequent. In addition, a description is given of the instruments used to measure each of them. For the analysis of reliability, two indices were used: Cronbach's Alpha (α) (equal to or greater than .70) [60], and Omega Coefficient (ω) [61], which also serves to check the internal consistency of the variables used in the investigation and, according to some authors (62), have shown evidence of greater accuracy. This means that in McDonald’s Omega Coefficient the established range is between 0 and 1, with the highest values giving us the most reliable measurements [62]. Structural validity was also examined through confirmatory factor analysis, respecting the criterion of eliminating those items with a regression weight that did not present an adequate value (greater than .40) [63].

#### Antecedent variables and measurement tools

Level of responsibility of the student during evaluation: In order to know the perception of the level of responsibility that is given to the student in the evaluation, The Scale of Student Responsibility during Evaluation in Physical Education (ERAEEF) was used, adapted to Spanish by Moreno et al. [64]. It is made up of 11 items divided into 2 factors. In the present study, the factor known as the value of the transfer of responsibility in the result of the evaluation was used composed of 5 items (E.g. Working with the PE teacher to decide my score is important). Results showed acceptable fit for reliability: α = .77, ω = .82; and for Confirmatory Factor Analysis (CFA) χ^2^ = 55.00, gl = 19, p < .001, χ2 / df = 2.89, CFI = .99, TLI = .98, RMSEA = .05 (CI 90% = .03, .06) [65].

#### Motivational variables and measurement tools

Basic Psychological Needs: To measure the satisfaction of the BPN, the Basic Psychological Needs in Exercise Scale (BPNES) was used, the original scale of Vlachopoulos & Michailidou [66] and validated to Spanish by Moreno et al. [67]. It is composed of 12 items divided into 3 factors. Each factor is made up of 4 items: Satisfaction of the BPN of Competence (E.g. The exercises that I perform are in line with my interests), Satisfaction of the BPN of Competence (E.g. I do the exercises effectively), Satisfaction of the BPN of Relatedness (E.g. I feel that I can communicate openly with my colleagues). Regarding the CFA, the results showed acceptable adjustment indices [65]: χ^2^ = 57.79, df = 24, p < .001, χ^2^ / df = 2.41, CFI = .99, TLI = .99, RMSEA = .04 (CI 90% = .03, .05).

Levels of Self-Determined Motivation: To measure the levels of self-determined motivation, the Perceived Locus of Causality in Physical Education (PLOC) was used. Original scale by Goudas et al. [68], and validated in Spanish by Moreno et al. [13]. It consists of 20 items divided into 5 factors. In the present study, a single factor has been used, autonomous motivation, composed of the grouping of intrinsic motivation (E.g. Because I enjoy learning new skills) and identified regulation (E.g. Because it is important for me to do well in PE). Regarding the CFA, the results showed acceptable adjustment indices [65]: χ^2^ = 296.79, gl = 62, p < .001, χ^2^ / gl = 4.35, CFI = .96, TLI = .97, RMSEA = .06 (90% CI = .05, .07).

#### Consequent variables and measurement tools

Satisfaction Level and Boredom in PE classes: To measure the level of satisfaction or boredom that students present in PE classes, the Sport Satisfaction Instrument (SSI) [69], validated to Spanish and adapted to Physical Education (SSI-EF) by Baena-Extremera et al. [70], was used. The SSI-EF was to Spanish. It is composed of 8 items divided into two factors, of which only satisfaction with PE classes was used, with 5 items (E.g. Normally I find PE interesting). Thus, in the Confirmatory Factor Analysis (CFA), the results showed acceptable adjustment indices [65]: χ^2^ = 5.29, df = 2, p < .001, χ^2^ / df = 2.65, CFI = .99, TLI = .99, RMSEA = .06 (CI 90% = .04, .09).

In all of the questionnaires that were used, answers were given to all of the items through a Likert Scale of 5 points, with a range from 0, which means the student is in complete disagreement, to 5, meaning that the student completely agrees.

### Procedure

Having defined the objectives of the study, the measurement instruments were selected in order to collect information, a dossier was prepared, and some interesting data was gathered, such as age, school year, the practice of extracurricular physical activity and the school to which the students belonged. Subsequently, the different schools were contacted and the objective of the study was explained. They were given a consent form for the parents to sign, as the students were under 18 years of age.

Following this, specific days were chosen for visiting the schools and handing out the questionnaires to those subjects with parent authorisation, never in the presence of the PE teacher. The time employed for the completion of the questionnaires was 40 minutes per class.

### Data analysis

First, we calculated mean, standard deviations, correlations and intraclass correlation (ICC) as indicator of non-independence. The study hypothesis was analysed through Structural Equation Modeling (SEM). Regarding the estimation method, taking into account that the answers of the participants were obtained through the Likert scale, and their answers are ordered categorically, we decided to use weighted least square mean and variance adjusted (WLSMV), as the estimation method, is more accurate than Maximum Likelihood [71], because they do not require multivariable or univariate normality [72].

It is important to note that data may not be independent, since students were nested within teachers. This nesting might lead to an overestimation of χ^2^ (e.g., worse fit) and underestimation of standard error (e.g., lower *p* values). To statistically correct it we used a sandwich-type estimator [73].

We used the delta method [74] to test the indirect effects of BPN and autonomous motivation in the relationship between the assignment of responsibility and satisfaction with the physical education classes.

Regarding the SEM, we used the following indexes of goodness of fit χ^2^ / gl, RMSEA (Root Mean Square Error of Approximation), CFI (Comparative Fit Index) and the Tucker-Lewis Index (TLI). The χ^2^ / gl is considered acceptable when it is lower than 5, the RMSEA with values lower than .05, and the CFI and TLI with values between .90 and .95 or higher, are considered as an acceptable to excellent fit [65, 75]. For descriptive analyses, the statistical program SPSS 21.0 was used, and for the SEM, calculations were done with Mplus 8.3 [76].

## Results

### Descriptive and reliability statistics

Table 3 presents the descriptive statistics, correlations and intraclass correlation of study variables. In terms of BPN, the highest mean value was for the need for relatedness, with the lowest average being the BPN of autonomy. With regard to ICC, classes satisfaction showed the highest value, while the BPN of relatedness the smallest.

**Table 3:** Means, standard deviations and correlations among variables.

Variable	Means	SD	ICC	1	2	3	4	5
1. Value Assignment	3.38	.93	.02	-				
2. BPN Autonomy	3.10	.96	.03	.33	-			
3. BPN Competence	3.68	.95	.02	.27	.55	-		
4. BPN Relatedness	3.98	.97	.01	.22	.38	.51	-	
5. Autonomous Motivation	3.68	.93	.03	.29	.60	.59	.38	-
6. Classes Satisfaction	3.89	.98	.09	.25	.60	.60	.44	.64

BPN, Basic Psychological Need; SD, Standard Deviation; ICC, Intraclass Correlation. All correlations were significant with *p* < .001

### Analysis of structural equations

In line with the HMM [21, 77], the antecedent variables (perception of the transfer of responsibility to the student in evaluation), the mediators (satisfaction of the BPN), self-determined types of motivation (autonomous motivation, controlled motivation and demotivation) and consequences (satisfaction with PE classes) were included.

**Fig 1:**
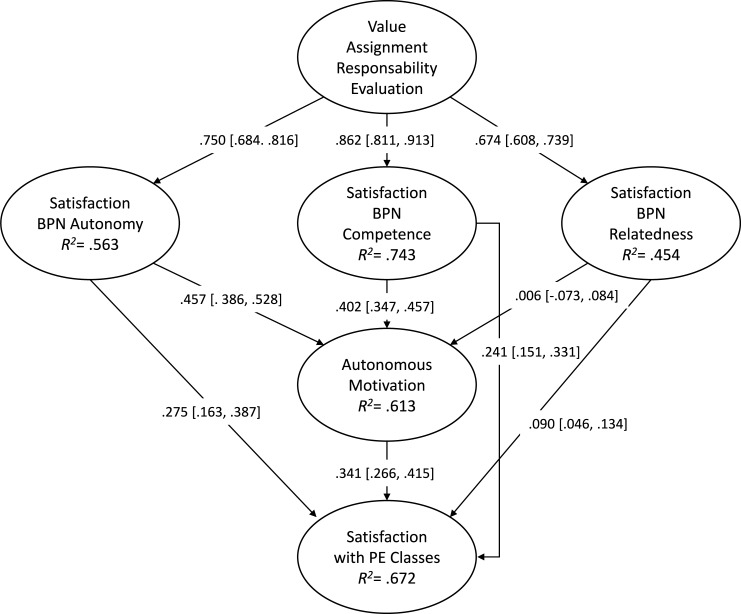
SEM. Predicting the students’ satisfaction with PE classes from assigning them with responsibility in evaluation and motivational variables. All of the parameters are standardised, the most statistically significant are indicated with *p < .01.

In this model, the aim was to find out the predictors of satisfaction with PE classes, based on the perception of assigning responsibility to students and the motivational variables (satisfaction of BPN and autonomous motivation). The results are shown in Fig 1.

The results of the structural equations model revealed a good fit to the data [65, 75]**: χ**^**2**^ **(921, 220) = 804.420 (*p* < .001),** χ^2^ / gl = 3.65, **RMSEA = .054 (CI 90% = .050, .056), CFI = .96, and TLI = .95.** The contribution of each factors to the prediction of other variables was examined through the standardised regression weights, hence, the value of the assignment of responsibility in the result of the evaluation predicted in a positive and significant way the satisfaction of the BPN of autonomy (β = .72 [.65, .79]), competence (β = .87 [.82, .92]) and relatedness (β = .70 [.63, .76]). On the other hand, autonomous motivation was predicted in a positive way by the satisfaction of the BPN of autonomy (β = .49 [.44, .54]), competence (β = .43 [.38, .48]) and relatedness (β = .04 [-.03, .11]), with self-motivation predicting in a positive and meaningful way the satisfaction with PE classes (β = .85 [.82, .88]).

Regarding the indirect effects between the latent variables, the indirect effect of BPN on the relationship between value assignment responsibility and autonomous motivation was .69 [.66, .73] and the indirect effect of BPN and autonomous motivation in the relationship between value assignment responsibility and satisfaction was .71 [.66, .76].

Table 4 shows the indirect effects. The indirect effects of the assigning of student responsibility in evaluation upon autonomous motivation vary according to the production level, via the satisfaction of the BPN of autonomy, which was β = .34 [.27, .42]. The satisfaction of the BPN of competence being β = .35 [.29, .40] and the satisfaction of the BPN of relatedness β = .00 [-.05, .06].

**Table 4:** Indirect effects in structural equation model.

Variables	Effects	*p*	CI
**V.** A. Responsibility → Autonomous Motivation	.69	< .001	.66, .73
** Via BPN autonomy**	.34	< .001	.27, .42
** Via BPN competence**	.35	< .001	.29, .40
** Via BPN relatedness**	.00	.89	-.05, .06
**V.** A. Responsibility → Satisfaction	.71	< .001	.66, .76
** Via BPN autonomy**	.21	< .001	.13, .28
** Via BPN competence**	.21	< .001	.13, .29
** Via BPN relatedness**	.06	< .001	.03, .09
** Via autonomous motivation and BPN autonomy**	.12	< .001	.08, .15
** Via autonomous motivation and BPN competence**	.12	< .001	.09, .15
** Via autonomous motivation and BPN relatedness**	.00	.89	-.02, .02

BPN, Basic Psychological Need; CI, Confidence interval; V. A., Value Assignment, p, significance index.

## Discussion and conclusions

Given the relevance of assigning responsibility to the student in the evaluation for improving motivational processes and increasing student satisfaction with PE classes, the present study aimed to validate a model that would analyse these relationships from the HMM.

The first hypothesis determined that “the assignment of responsibility towards the student will predict, in a positive and significant way, the satisfaction of the BPN of autonomy, competence and relatedness”. In terms of the results previously mentioned, it can be observed that the hypothesis is fulfilled, since the results showed that the perception of the assignment of responsibility to the student in the evaluation predicted in a positive and significant way the satisfaction of the BPN (autonomy, competence and relatedness).

Some studies have demonstrated that when students are offered the opportunity to choose tasks they improve their skills, their physical activity and their perceived competence [78]. It was also proven that there was a greater learner involvement when given the opportunity to make decisions with various methodological aspects such as space, time, material or grouping [79].

In line with results obtained, Moreno et al. [80] showed that responsibility positively predicted psychological mediators. Research carried out by Vera [26], with 49 students, also showed that the assignment of responsibilities to the students makes the satisfaction of the BPN of autonomy higher, and with it the satisfaction and enjoyment towards physical activity.

The study by Gómez-Rijo et al. [81], reached the conclusion that the transfer of responsibilities to the student, by the teacher, contributes to the development of student autonomy.

Motivation involves a set of emotional, cognitive and social phenomena, with which, according to studies, if a teaching style is used where students are allowed to participate in the teaching-learning process, the cognitive and physical involvement will be greater [82]. This explains a greater satisfaction towards PE classes, and a greater commitment to learning, as students are more intrinsically motivated thanks to their involvement in the evaluation process [31].

The second hypothesis exposed that “the satisfaction of the BPN of autonomy, competence and relatedness will predict, in a positive and significant way, the autonomous motivation”. In terms of the results previously mentioned, it can be observed that the hypothesis is fulfilled, since positive and significant relationships between these variables were found.

Moreno et al. [80], in addition to showed that responsibility positively predicted psychological mediators, also showed that this predicted intrinsic motivation, which positively predicted the importance that students give to physical education, and this, finally, positively predicted the student's intention to continue playing sports.

It has been confirmed that one key aspect to improving motivation is the assignment of responsibility to the student [18], along with the use of styles that favour the autonomy of the students [83]. Thanks to different works [18, 38], which are in line with our results, it can be affirmed that an assignment of responsibilities increases the most self-determined forms of motivation. It was also demonstrated [84] that giving autonomy to the student for the learning of physical skills improves autonomous motivation.

Evaluation must not only be linked to the teacher giving a score, but the student must be given the possibility to decide and intervene, taking into account initial and bidirectional agreements [85]. Other works developed in the educational field [86, 87], which related the satisfaction of the BPN to the self-determined forms of motivation, revealed, like in our study, that an adequate satisfaction of the BPN would increase intrinsic motivation.

The authors of these works have shown that a greater feeling of autonomy will increase intrinsic motivation [8, 9, 49, 57, 88, 89], supporting the results found in this study. However, other authors, performing intervention programmes to support teachers with the BNP, did not find significant results in intrinsic motivation [90].

Research that is also related to our variables [44, 48, 51, 52], indicated that the satisfaction of the BPN predicted high levels of intrinsic motivation and that this was related to an increase in enjoyment and satisfaction with classes. It can therefore be said that there is a close relationship between intrinsic motivation and satisfaction with classes [91, 92]. Some authors, in their results give particular importance to the BPN of autonomy [58, 93].

Finally, the third hypothesis raised that “the autonomous motivation will predict, in a positive and significant way, student satisfaction with PE classes”. After an analysis of data, positives and significant relationship were found, between BPN of autonomy, competence and relatedness with student satisfaction with PE classes directly, and no mediated through autonomous motivation, as has been raised in the third hypothesis. Therefore, we reject compliance with this last hypothesis.

As in our results, the studies confirm the importance of LBW for the creation of positive consequences in PE classes [50, 94]. However, there are not many studies that directly relate BPN with positive consequences, without mediating autonomous motivation, as in the present study.

Similar to our results, Curran & Standage [95] confirmed a direct relationship between the satisfaction of the three BPN of the students and their commitment to PE classes. In the same line Burchard et al. [96], found that the satisfaction of the three LBW in the PE classes was directly related to the overall self-esteem of the adolescents. Also, Franco et al. [97], with PE students performing a SEM, obtained a direct relationship between the BPN of competence and self-esteem. Likewise, Di Battista et al. [98], found a direct relationship between the BPN of competence and the intention to be physically active, in PE students.

However, in the present study we found that the BPN of autonomy is the BPN that has more predictive power on satisfaction with PE classes. There are several studies that confirm the relation of the BPN of autonomy with the satisfaction and / or enjoyment in PE classes, although mediated by autonomous motivation [35, 36, 37, 91, 99] and not directly, as in our results.

Other studies which had similar results to ours, indicate that an increase in autonomy will make satisfaction with PE classes higher [79], and that students will be more involved in their tasks and their own learning process [79]. Different investigations, such as the one carried out by Méndez et al. [100], found that if a suitable atmosphere that involves the task is generated in the classroom, the satisfaction of the BPN will be greater, which will be positively related to more self-determined motivation and with less boredom with the classes of PE.

A teaching-style where autonomy and decision-making is stimulated will reduce the demotivation of the students, as well as boredom with PE classes [100], as pointed out in the study by Moreno et al. [101]. Different works have indicated that the less self-determined forms of motivation and a lower perception of satisfaction of BPN [102] are related to the giving up of physical activity, which may be due to the lack of satisfaction with the PE classes.

As many researchers have been proposing for some time [32, 103, 104], it is necessary to come up with new evaluation strategies, which offer more student involvement. Strategies such as developing the students’ ability to reflect on what they have done, substitute the final exam for a continuous process in which the students learn from their mistakes and successes, involve the student in making decisions, among others, which will mean that, based on the theoretical postulates of the HMM, the satisfaction of the BPN will be higher, as well as that the more self-determined forms of motivation will be increased, with levels of demotivation decreasing. This will have positive consequences, such as satisfaction with PE classes, and therefore, increase the possibility of physical activity outside the classroom.

One of the limitations found in this study was the sample, which would be interesting to expand to other areas and even differentiate by age, gender and socioeconomic level. Another limitation was seen from only using questionnaires, as only opinion is determined through a scale of answers. It would be interesting to make a methodological triangulation, using systematic observation and the use of interviews, both with students and with teachers. Once the results are known, a longitudinal or quasi-experimental study could be carried out, through an intervention that would allow us to establish cause-effect relationships, in order to know the effect caused by the application of different motivational strategies in the variables under analysis.

In conclusion, thanks to results from models such as ours, in PE classes intervention programmes are necessary to achieve more self-determined motivation of the students, through the BPN, especially the BPN of autonomy and competence. This can be done using different strategies, such as reciprocal evaluation, so that they feel they are participants in their teaching and learning process, proposing self-evaluation activities, as well as posing tasks that are fun for them, by assigning them with responsibilities; teachers should place special emphasis on the use of strategies where individualised activities are proposed, that represent an achievable challenge for the students. In this way, teachers can achieve that students increase their levels of satisfaction of the BPN, which will lead to them showing higher levels of autonomous motivation in the classes and a greater satisfaction with the PE classes.

## Supporting information

S1 File DATA_for_R_and_MPLUS. (SAV)

S2 File 20.6.2019. (CSV)

S3 File Variables names. (PDF)
