# Supplementary material for: The importance of assigning responsibility during evaluation in order to increase student satisfaction from physical education classes: A structural equation model
Source: PLoS One. 2019 Sep 10;14(9):e0209398. doi: 10.1371/journal.pone.0209398 (PMC6736274; doi:10.1371/journal.pone.0209398)
Supplement: S3 File — (PDF) [file pone.0209398.s003.pdf]

Name given to each variable for the MPlus:

| **Name Mplus** | |
|---|---|
| | **PLOC** |
| MAUTO | Autonomous Motivation |
| | **BPN** |
| AUTON | Autonomy |
| COMPE | Competence |
| RELAC | Relatedness |
| | **ERAEEF** |
| VALOR | Value of the transfer of responsibility in the result of the evaluation |
| | **SSI-EF** |
| SAT | Satisfaction |
